# The Relationships of Watching Television, Computer Use, Physical Activity, and Food Preferences to Body Mass Index: Gender and Nativity Differences among Adolescents in Saudi Arabia

**DOI:** 10.3390/ijerph18189915

**Published:** 2021-09-21

**Authors:** Ahmad H. Alghadir, Zaheen A. Iqbal, Sami A. Gabr

**Affiliations:** Department of Rehabilitation Sciences, College of Applied Medical Sciences, King Saud University, Riyadh 11433, Saudi Arabia; aha@ksu.edu.sa (A.H.A.); dr.samigabr@gmail.com (S.A.G.)

**Keywords:** screen-time, obesity, physical activity, BMI, adolescent health, online teaching, COVID-19 pandemic lifestyle

## Abstract

Background: Adolescents and ethnic subgroups have been identified at high risks of overweight and its associated complications. Although some studies have investigated overweight, obesity, nutritional status, physical activity, and associated factors among Saudi students, no studies have examined these characteristics among non-Saudi students or compared non-Saudi to Saudi adolescent students. The objective of this study was to compare differences between Saudi and non-Saudi adolescent students regarding time spent watching television, using computers, engaging in physical activity, and their food preferences. The relationships between these lifestyle behaviors and body mass index by Saudi nativity and gender were tested. Methods: Students aged 12 to 18 years (*n* = 214) from various schools in Riyadh, Saudi Arabia, completed a self-administered questionnaire that included questions about demographic and anthropometric characteristics, daily after-school routine, physical activity, watching television, using computers, and food preferences. Non-parametric (Mann–Whitney U) tests assessed the statistical differences between Saudi and non-Saudi respondents, and males and females were separately tested. Results: Saudi boys who reported physical activity two to five times per week, the most television time, the most computer time, and the highest frequency of eating fast food and drinking soft drinks had a significantly higher mean body mass index than the non-Saudi boys in their categories. However, there were no significant differences found between the Saudi and non-Saudi girls. Conclusions: High levels of sedentary and low levels of physical activities as well as high consumption of high-fat fast foods and high-sugar drinks threaten the health of Saudi adolescents. Cultural differences in lifestyle between Saudi and non-Saudi families should be considered when developing programs to improve knowledge, attitudes, and behaviors regarding diet quality and physical activity. The objective of this study is more important in the current situation where increased time spent on computers and mobile phones due to online teaching in schools or working from home, decreased physical activity due to precautionary lockdowns, and unchecked eating patterns while spending more time in sedentary activities in homes has become our COVID-19 pandemic lifestyle in all the age groups. A similar study should be replicated on a large scale to study the effect of this lifestyle on our lives in all the age groups.

## 1. Introduction

Adolescents and ethnic subgroups have been identified as having high risks of overweight and its associated complications [1,2,3], and overweight at young ages has strongly predicted overweight and its associated morbidity and mortality during adulthood [4,5]. Dramatic changes occur in health-related behaviors during adolescence [6], including changes in eating habits, and decisions about, and the length of time, spent in sedentary or physical activities [7]. Such unhealthy habits developed during adolescence have been found to persist into later life stages [8]. Long periods of sedentary behaviors and short periods of physical activity combined with consumption of high-calorie foods play a major part in weight gain during adolescence [9]. Among the various sedentary activities with limited energy expenditure, such as computer use, reading, playing video games, and driving a car, extensive television watching has been widely reported as a risk factor for overweight and obesity [10,11]. Usage of computers and mobile phones has further increased these days as most of the schools have adopted online teaching methods amid the COVID-19 pandemic [12]. Prolonged use of computers and mobile phones have been shown to cause neck and back pain and altered body posture including forward head posture across all age groups [13,14].

Socioeconomic status and ethnicity have been associated with each other [15]. Obesity and its associated complications might be relatively prevalent in some ethnic groups because of their cultural characteristics expressed in everyday lifestyles and habits [16,17,18]. Various previous studies from the UK, US, and Europe have indicated that behavioral differences related to ethnicity might lead to overweight during adolescence [6,19,20] and studies of adolescents from various ethnic backgrounds have found that some sedentary and physical activities associated with unhealthy diet patterns differently influenced body mass index (BMI) [21,22,23]. The results of these studies have been used to devise preventive strategies to prevent adolescent obesity [24,25].

Saudi Arabia is a rapidly developing country recently undergoing important demographic and public health changes [26,27]. Lifestyle modernization is decreasing the average amount of the average Saudi’s physical activity [28,29]. Some research has found that, on average, about 34% of Saudi university students are overweight [30,31]. Although things are changing now, Saudi culture was considered to be relatively closed till recently. Females were not allowed to drive a car and all the girls had to wear a special dress before going out of their homes. In some places, they were not even allowed to go out of their homes without an accompanying male guardian. Such rules are not prevalent in the cultures in other countries, especially from where expatriates come from. These rules can have both negative and positive effects, specifically among adolescents. The kingdom’s development and growth is attracting large number of skilled and unskilled workers from around the world, most of whom immigrate with their families. According to the Central Department of Statistics and Information, in 2014, about 33% (about 10.1 million people) of the population was immigrants [32].

Although some studies have investigated overweight, obesity, nutritional status, physical activity, and associated factors among Saudi students, to the best of our knowledge no studies have examined these characteristics among non-Saudi students or compared non-Saudi to Saudi adolescent students. Consequently, this study compared a sample of Saudi to non-Saudi adolescent students regarding the amount of time spent on physical activities, watching television, computer use, and their food preferences. The study’s main objective was to compare differences between Saudi and non-Saudi adolescent students regarding time spent watching television, using computers, engaging in physical activity, and their food preferences. The relationships between these lifestyle behaviors and body mass index by Saudi nativity and gender were tested. Our results are intended as a foundation for devising preventive strategies for adolescent obesity.

## 2. Materials and Methods

### 2.1. Sampling and Data Collection

Children aged 12 through 18 years old attending various schools in Riyadh were invited to participate in this study. A self-administered questionnaire was used to collect the data. The study’s purposes and goals were explained to the adolescents and their parents and their written consent was obtained before the survey was administered. They were assured of the confidentiality of all the information they provided for the study. Questionnaires with missing data and respondents with any type of disability were dropped from the analysis. The institutional review board approved all procedures performed in accordance with the Helsinki Declaration for ethical standards of human research.

### 2.2. Variables and Measurement

The questionnaire included the questions about demographic and anthropometric characteristics, daily after-school routine, physical activity, watching television, using computers, and food preferences [21,26,33,34,35]. Their weight (kg) and height (cm) were measured to the nearest 0.1 kg using a digital metric scale, and to the nearest 5 mm using a wall-mounted height board, respectively, in the school medical room. BMI was subsequently calculated as weight divided by height square (kg/m^2^) [36,37,38,39]. Waist circumference (cm) was measured as the minimum circumference between the hip bone and the rib cage [40].

Respondents were asked to assess the average time per day they spent while watching television or using computers. Television viewing (h/day) time included time spent watching television, videos, or DVDs. Computer use (h/day) time was defined as the time spent while using a computer, laptop, or playing video games. They were also asked to report the number of times per week (times/week) during which they participated in any physical activity enough to cause sweating. Section on food preferences included questions about breakfast, daily milk consumption, taking home made lunch to school, and frequency (times/week) of consuming high-fat fast foods and high-sugar soft drinks.

### 2.3. Analysis

Graph-Pad Instat 3.0 (GraphPad Software, San Diego, CA, USA) was used to analyze the data. Non-parametric (Mann–Whitney U) tests were used to assess the statistical differences in BMI between Saudi and non-Saudi respondents in relation to various parameters including physical activity, watching television, computer use, and food preferences. Differences were considered statistically significant at *p* < 0.05.

## 3. Results

Of the 300 students who agreed to participate in the study, 250 (83%) returned the questionnaires. Of them, 36 respondents did not meet the inclusion criteria, and the final sample comprised 214 (71%). Table 1 shows the respondent’s personal characteristics by gender and nativity. The mean age of respondents was 14.41 years (SD = 2.36). About 46% of the sample was Saudi. The non-Saudi respondents were from Australia, India, Pakistan, Afghanistan, South Africa, or Egypt. At the time of the survey, 35% of the sample was in secondary school and the rest were at the pre-university level.

About 89% (*n* = 190) of the respondents reported that at least one parent suffered from diabetes, heart disease, or another health problem; 88% (*n* = 189) reported that they never smoked cigarettes, and about 83% (*n* = 177) considered themselves fit and healthy compared to their friends.

### 3.1. BMI and Lifestyle Differences

The mean BMIs of the Saudi and non-Saudi respondents were 24.02 kg/m^2^ (SD = 1.34) and 24.21 (SD = 4.34), respectively, which was not significantly different. However, the difference between Saudi (24.51) and non-Saudi (23.74) boys was statistically significant *p* < 0.05 (Table 2).

Overall 8% of the 214 respondents reported that their favorite after-school activity was playing outdoor games, such as football. The other respondents reported that their favorite activities were using the internet or playing computer games. For their transportation to and from school, 67, 26, and 7% used a car or bus, walked, or cycled, respectively. Table 3 and Table 4 report the lifestyle differences and their relationships to mean BMI between the Saudi and non-Saudi boys and girls, respectively.

### 3.2. Watching Television

About 87% of the Saudi boys and about 69% of the non-Saudis boys reported watching television more than two hours per day. The difference in mean BMI between the Saudi and non-Saudi boys who watched the most television per day was statistically significant (Saudi = 24.57 vs. non-Saudi = 23.67, *p* < 0.05). High proportions of the girls also reported watching television more than two hours per day, (Saudi = 76% and non-Saudi = 63%), but the association between watching television and mean BMI was not significantly different for the girls.

### 3.3. Computer Use

Amongst all of the respondents, 94% reported to ownership of a personal laptop or a desktop computer at home, and 85% of the overall sample reported computer use of more than two hours per day. Among the boys, 98%of the Saudis and 80% of the non-Saudis and, among the girls, 89% of the Saudis and 74% of the non-Saudis reported computer use of more than two hours per day. Among the boys with more than two hours per day of computer use, the non-Saudis had significantly lower mean BMI (Saudi = 24.52 vs. non-Saudi = 23.61, *p* < 0.05).

### 3.4. Physical Activity

Among the boys, almost all of the Saudis (98%) and most (80%) of the non-Saudis reported physical activity (enough to cause sweating) two to five times per week. Similarly, among the girls, 98% of the Saudis and 73% of the non-Saudis reported two to five days of physical activity per week. There was a significant difference in BMI among the boys, but not among the girls, at this activity level (Saudi = 24.52 vs. non-Saudi = 23.70, *p* < 0.05).

### 3.5. Food Preferences

Overall, the majority of the respondents 81% reported eating breakfast daily, 36% reported not drinking milk every day, and about 45%of the respondents did not take a lunch to school and they either skipped lunch or ate purchased food.

### 3.6. High-Fat Fast Foods and High-Sugar Soft Drinks

Just 3% (*n* = 7) of the respondents reported that they did not like to eat high-fat fast foods and preferred to eat at home. Similarly, just 6% (*n* = 12) of the respondents reported not liking high-sugar soft drinks.

Among the Saudi boys, those who consumed high-fat fast foods were overwhelmingly likely to do so more than three times per week (97%). On the other hand, 28% of the non-Saudi boys ate these foods once, twice, or three times per week and 67% of them ate them more than three times per week. Comparing the boys who ate high-fat fast foods the most often, the non-Saudi boys had a significantly lower mean BMI (Saudi = 24.47 vs. non-Saudi = 23.73, *p* < 0.05). The consumption patterns of the Saudi and non-Saudi girls were similar, but the Saudi girls were less likely than their male counterparts to consume these foods more than three times per week (Saudi girls = 79% vs. Saudi boys = 97%), and the difference in mean BMI between the Saudi and non-Saudi girls was not statistically significant at any consumption level.

Regarding high-sugar soft drinks, 83% of the Saudi and 67% of the non-Saudi boys consumed them most frequently (more than three times per week), and their mean BMIs were different (Saudi = 24.49 vs. non-Saudi = 23.95), *p* < 0.05). The girls also were most likely to consume soft drinks at the most frequent level (Saudi = 83% and non-Saudi = 62%) but the difference in mean BMI among those girls (Saudi = 23.88 vs. non-Saudi = 25.34) was not significantly different.

## 4. Discussion

This study compared Saudi to non-Saudi adolescent students regarding watching television, computer use, physical activity, and food preferences. To the best of our knowledge, only a few studies have also examined cross-cultural differences between Saudi and non-Saudi adolescent students. Saudi boys who reported the highest levels of television and computer use, moderate physical activity, and the highest frequency of fast food and soft drink consumption had significantly higher mean BMIs than the non-Saudi boys at those levels.

A further comparison of the Saudi and non-Saudi respondents regarding sedentary and physical activities and food preferences found that mean BMI was partly influenced by ethnic differences. Ethnic differences in body composition mediated by energy-balance related behaviors (intake and expenditure) were found in studies set in other countries, such as Germany and the Netherlands [8,41].

Increased time spent on computers and mobile phones due to online teaching in schools or work from home, decreased physical activity due to precautionary lockdowns, and unchecked eating patterns while spending more time in sedentary activities in homes has become the COVID-19 pandemic lifestyle in all the age groups. Although this study was conducted before the beginning of theCOVID-19 pandemic, its objective has strong relevance in the current situation. A similar study should be replicated on a large scale to study the effect of the COVID-19 pandemic lifestyle on our lives in all the age groups.

One of the previous studies has shown that body composition indices and sitting time associated with media use were higher among Saudi boys and that expatriate boys and girls were physically more active compared to Saudis of the same age group [36]. Our results also show that Saudi boys had a higher mean BMI than non-Saudi boys that might be explained by the longer time period spent watching television, using computers, and their less frequent physical activity. For example, none of the Saudi boys watched television for less than one hour per week as compared to 14% of the non-Saudi boys, and none of them were physically active more than five days per week compared to 7% of the non-Saudi boys. The Saudi boys also reported more frequent consumption of high-fat fast foods and high-sugar soft drinks than the non-Saudi boys. Previous studies reported that the amount of time spent watching television was related to overweight and obesity [26,42,43], and the amount of time spent in sedentary activities negatively influenced body composition more than medium or high intensity physical activity positively influenced it [44]. This is more important in the current situation when physical activities of students have decreased due to precautionary lockdowns while schools have started online teaching that has considerably increased sedentary activities, including time spent on computers and mobile phones.

Cycling or walking to or from school was previously found to significantly influence BMI [9,45,46]. The majority of the respondents in the current study reported traveling to school either by car or school bus. Longer travel time to and from school using these means necessitates sitting for periods found to be an important influence on student’s nutritional status [47] and this might be a reason for the high prevalence of overweight Saudi students. Differences in BMI between ethnic groups with similar daily activities and food preferences might be due to interactions between environmental and social factors that affect individuals’ decisions about physical activity and food preferences [11,48]. Saudi Arabia’s extreme dry climate is another factor that might reduce students’ physical activities. It has been reported that students should be encouraged to increase their physical activity and eat a healthy balanced diet that includes Vitamin E to improve academic performance and executive function [49]. Optimal daily physical activity has also been regarded as a determining factor for academic performance [37].

The vast majority of our respondents (94%) reported owning a personal laptop or desktop computer at home. Personal television sets, computers, laptops, tablets, and so on in children’s bedrooms are key reasons for their long periods of screen time. Watching television might indirectly increase food consumption while reducing physical activity and energy expenditures [33,50] and watching television while eating has been identified as a risk factor for obesity [51]. Television advertisements for fast foods also have been found to influence eating behaviors, particularly among children [22,52].

Some studies have indicated that the amount of time spent engaged in sedentary activities, such as watching television, during childhood and adolescence predicts overweight and obesity in adulthood [44,53,54]. This link was identified in a review prepared for the World Health Organization [55,56], and, based on those studies, decreasing children’s television time was recommended [23]. It is important to note that the influences of television time on health might differ across cultures and by the nature of the viewed content [22].

High consumption levels of sweetened drinks and snack foods have been associated with weight gain among adults and children [57,58]. However, because genetic metabolic rates might be ethnicity specific, individuals with similar physical activities and diet preferences might have different risks of weight gain. The timing and frequency of meals also might influence that risk [59].

Imbalances between proportions of artificially sweetened drinks and fatty fast foods on the one hand and fresh fruits, vegetables, and dairy products on the other hand influence children’s health [60]. Individuals’ eating habits also are influenced by social and lifestyle factors [22]. Our results supported the findings of a previous study conducted in Saudi Arabia that found 30% of the boys and 21% of the girls ate fast foods [30] and another study reported a high rate of energy drink consumption (56% of the boys and 35% of the girls) [61]. A 260 mL soft drink provides about 188 kcal of energy [62], which, if not counteracted by physical activity, is stored as fat and results in weight gain [63].

Although no direct links have been established between the composition of consumed food and weight gain among adolescents, eating late at night, omitting breakfast, and frequent snacking between meals have been found to variously influence individuals [64]. In one study, the majority of the respondents (81%) reported not omitting breakfast because breakfast is the most important meal of the day [65]. Although eating breakfast increases the total number of daily calories consumed, the likelihood of weight gain is less among those who eat breakfast [66,67] and their cognitive and memory functions are better [68,69]. In our study, about 36% of the respondents reported not drinking milk daily, and 45% of the respondents did not take a lunch to school. The importance of milk, fresh fruit, and home-cooked food has been widely reported [63,70]. Thus, parents should insist that their children at least one meal each day with the family and without television. When parents eat with their children, the children tend to have healthier diets [71]. People have adopted various bad habits related to eating patterns such as eating late at night, omitting breakfast, frequent snacking between meals, etc. during the current pandemic situation. This, associated with decreased physical activities and increased sedentary activities, would affect our bodies badly. This needs to be studied further in ordered to prevent associated consequences.

Interestingly, there were no significant differences in mean BMI between the Saudi and non-Saudi girls within the lifestyle categories of the variables. The pattern of differences between the Saudi and non-Saudi girls was very similar to the pattern of differences between the two groups of boys. Compared to the non-Saudi girls, the Saudi girls were relatively likely to watch the most number of hours of television and spend to most number of hours on a computer per day (>2), to have low levels of physical activity (<2 times per week), and to consume fast foods and soft drinks more than three times per week. The finding is not consistent with previous studies that found direct relationships between preferences for high-fat and high-sugar content foods and overweight among girls [72]. In our study, the majority of the non-Saudi respondents were from Egypt. Some studies have reported similar lifestyles and food preferences among Gulf Cooperation Council and their neighboring countries [73,74], which might partly explain our non-significant results for the girls.

Since the studied age group is more active on various social media platforms, promotion of healthy lifestyle including food habits and benefits of physical activity can help prevent associated risk and complications. We also recommended that the political authorities should implement various measures and protocols to alleviate the potential risk related to unhealthy lifestyles. Age difference between 12 to 18 years is too large. We propose that future studies should divide the age by groups and be conducted on a larger sample size to get better clarification about the variables. Various studies have associated the social, economical, and educational levels of parents, especially mothers, in the health status among children. We propose that this should be considered in similar future studies.

### Limitations

The current study did not include equal representation of Saudi and non-Saudi children. It used a self-report questionnaire and the respondents might have exaggerated or under-reported their activities. Girls in adolescence often use various weight control diets, which could have affected the results of this study.

## 5. Conclusions

High levels of sedentary and low levels of physical activities as well as high consumption of high-fat fast foods and high-sugar drinks threaten the health of Saudi adolescents. Cultural differences in lifestyle between Saudi and non-Saudi families should be considered when developing programs to improve knowledge, attitudes, and behaviors regarding diet quality and physical activity. The objective of this study is more important in the current situation where increased time spent on computers and mobile phones due to online teaching in schools or working from home, decreased physical activity due to precautionary lockdowns, and unchecked eating patterns while spending more time in sedentary activities in homes has become our COVID-19 pandemic lifestyle in all the age groups. A similar study should be replicated on a large scale to study the effect of this lifestyle on our lives in all the age groups.

## Figures and Tables

**Table 1:** Gender based demographic and anthropometric characteristics of the Saudi (46%, *n* = 99) and non-Saudi (54%, *n* = 115) respondents.

Characteristic	Boys		Girls	
	Saudi(*n* = 52)	Non-Saudi (*n* = 72)	Saudi(*n* = 47)	Non-Saudi (*n* = 43)
Age (in years)	13.32 (1.98)	15.69 (2.16)	12.91 (1.13)	15.23 (2.58)
Height (cm)	168.03 (4.82)	167.73 (20.51)	167.27 (2.79)	163.30 (10.82)
Weight (kg)	69.19 (4.09)	68.83 (8.93)	66.76 (3.25)	67.82 (16.06)
Waist circumference (cm)	100.88 (15.80)	90.29 (39.22)	97.29 (8.88)	85.83 (30.69)

**Table 2:** Gender based body mass index (kg/m^2^) of the Saudi (46%, *n* = 99) and non-Saudi (54%, *n* = 115) respondents.

Group	Body Mass Index (BMI)	
	Mean	Standard Deviation
**Boys**		
Saudi (*n* = 52)	24.51 *	1.29
Non-Saudi (*n* = 72)	23.74	2.18
**Girls**		
Saudi (*n* = 47)	23.87	1.32
Non-Saudi (*n* = 43)	24.99	6.49

* *p* < 0.05.

**Table 3:** Nativity based variations in lifestyle behaviors (watching television, computer use, physical activity, and food preferences) and their relationships to mean body mass index (BMI) among boys.

Variable	Nativity			
	Saudi (*n* = 52)		Non-Saudi (*n* = 72)	
	*n* (%)	Mean BMI(SD)	*n* (%)	Mean BMI (SD)
Watching television (h/day)				
<1	0 (00)	00 (00)	10 (14)	23.64 (4.05)
1–2	7 (13)	24.10 (0.78)	12 (17)	23.82 (2.63)
>2	45 (87)	24.57 (1.35)	50 (69)	23.67 (1.63) *
Computer use (h/day)				
<1	0 (00)	00 (00)	7 (10)	23.83 (3.06)
1–2	1 (02)	24.09 (00)	8 (11)	24.52 (3.73)
>2	51 (98)	24.52 (1.30)	57 (79)	23.61 (1.78) *
Physical activity (times/week)				
<2	1 (02)	24.22 (00)	9 (13)	24.12 (3.01)
2–5	51 (98)	24.52 (1.30)	58 (80)	23.70 (1.95) *
>5	0 (00)	00 (00)	5 (7)	23.50 (3.37)
Consumption of high-fat fast foods (times/week)				
Zero	0 (00)	00 (00)	4 (6)	22.91 (1.44)
1–3	2 (03)	25.49 (0.82)	20 (28)	23.931 (3.40)
>3	50 (97)	24.47 (1.30)	48 (67)	23.73 (1.51) *
Consumption of high-sugar soft drinks (times/week)				
Zero	0 (00)	00 (00)	6 (8)	24.87 (2.97)
1–3	9 (17)	24.59 (1.18)	18 (25)	22.79 (3.35)
>3	43 (83)	24.49 (1.33)	48 (67)	23.95 (1.27) *

* *p* < 0.05.

**Table 4:** Nativity based variations in lifestyle behaviors (watching television, computer use, physical activity, and food preferences) and their relationships to mean body mass index (BMI) among girls.

Variable	Nativity			
	Saudi (*n* = 47)		Non-Saudi (*n* = 43)	
	*n* (%)	Mean BMI (SD)	*n* (%)	Mean BMI (SD)
Watching television (h/day)				
<1	3 (6)	23.14 (1.32)	11 (26)	24.30 (4.40)
1–2	8 (17)	24.10 (1.45)	5 (12)	23.25 (1.89)
>2	36 (76)	23.88 (1.30)	27 (63)	24.38 (1.56)
Computer use (h/day)				
<1	0 (00)	00 (00)	3 (7)	22.86 (2.85)
1–2	5 (11)	24.36 (1.85)	8 (19)	25.99 (4.21)
>2	42 (89)	23.81 (1.26)	32 (74)	24.94 (7.20)
Physical activity (times/week)				
<2	1 (2)	21.22 (00)	7 (18)	24.31 (3.26)
2–5	45 (98)	23.95 (1.28)	31 (73)	24.62 (2.63)
>5	1 (2)	23.03 (00)	5 (9)	22.25 (2.81)
Consumption of high-fat fast foods (times/week)				
Zero	0 (00)	00 (00)	3 (7)	23.79 (3.71)
1–3	10 (21)	24.21 (1.17)	17 (40)	26.67 (10.09)
>3	37 (79)	23.78 (1.35)	23 (53)	23.91 (1.21)
Consumption of high-sugar soft drinks (times/week)				
Zero	0 (00)	00 (00)	6 (17)	23.39 (2.24)
1–3	8 (17)	23.82 (1.12)	11 (26)	25.04 (3.74)
>3	39 (83)	23.88 (1.37)	26 (62)	25.34 (7.96)

## Data Availability

The datasets used in this study are available from the corresponding author on request.
